# Reflections

**Published:** 1885-11

**Authors:** Ad. Lippe

**Affiliations:** Philadelphia


### REFLECTIONS.

AD. LIPPE, M. D., PHILADELPHIA.

There is before us a new work emanating from the Hahnemann Publishing House, Philadelphia. The title-page of this work reads, *A System of Medicine Based upon the Law of Homœopathy*, edited by H. R. Arndt, M. D. Upon reflection this title-page is quite an enigma. Is it a *new* system of medicine growing out of or based upon the laws governing Homœopathy, or what can it really be? Is there really a Law of Homœopathy? or is not Homœopathy, as promulgated and taught by its founder, Samuel Hahnemann, an art which teaches how to apply the only possible law of cure—the law of the similars—to the science of therapeutics? An explanation of this healing art was given by its founder in his great philosophical work, *The Organon of the Healing Art.* The only possible solution must be, upon sober and calm reflection, that this new work expounds, upon the basis of the laws governing homœopathic laws of therapeutics, a progressive system superseding Hahnemann's methods, or else that it is a fraud. The title-page gives no satisfactory explanation, and we now proceed to reflect upon the new revelations to be found in the preface. There the editor tells us that he possesses one great advantage over the majority of the progenitors of new books, in that he need not enlarge upon the *raison d'etre* of this publication. The entire homœopathic school have for years experienced the want of a work on "Practice," which should take the place of the very excellent, but now old, treatises of Baehr, Kafka, and others. It is, after all, *not* a new system of medicine; it is a work on "Practice" wanted by the entire homœopathic school! If the editor had said, "by a majority of *professedly* homœopathic practitioners," he would probably have been right in his assertion ; BUT there is still the old guard, the pioneers of our school, who *know well* that all the works on Practice, from the *Domestic Physicians* down to Kafka's work were, and had to be, *failures*. They were and are progressive attempts to generalize ; they are an impossibility, for the reason that in each individual case the true healer is compelled to individualize. The editor further says (page 8) : "In each instance, then, the reader has placed before him the result of actual experience gained by observation obtained at the bedside, a feature of the work which becomes of great importance in those portions which deal with the *treatment* of special pathological conditions ;" and on page 9 the editor further confirms his position when he says : "The indications for remedies are of necessity given with reference only to symptoms which occur in direct connection with disorders treated ; concomitant symptoms usually are ignored, because a work like this cannot be made to take the place of a work on symptomatology."

Does the editor of this work ignore the well-known fact that the homœopathic healing art *never* did and never can treat special pathological conditions, or does he imagine that a treatment of special pathological conditions will bring satisfactory results ? What are special pathological conditions ? What is sick physiology ? It always has been, as Hahnemann so lucidly described them, merely a hypothesis, a misleading, speculative guess-work, and so it will be to the end of the world. Concomitant symptoms usually are ignored in this work. What, then, is the true inwardness of this new system ? It is a modern attempt to put the pathological livery on the homœopathic healing art. Valuable material has been collected by Ruckert and Oehme giving the experiences gained by observations at the bedside, under the title *Ruckert's Clinical Observations*. Ruckert or any of the collectors of such clinical observations never claimed to make them a basis of a system of medicine. These five volumes have been valuable to the student of Homœopathy. Concomitant symptoms were not ignored, and there was no attempt made to generalize. Pathology as a collateral branch of the medical sciences has never been ignored by the true healer as a means of coping with the allopathists. They make their pathology the basis of therapeutics and we do not ; all the attempts to reduce Hahnemann's methods to the adaptation of the allopathic unsuccessful attempt will fail. Any work written for that purpose will fail, as it is misleading, unreliable, a false guide. Let us reflect for a moment on the text of the book, and we just open Vol. I, page 464—*Angina pectoris.*

There is a better and a decidedly more *honest* essay to be found on *Angina pectoris* in the fourteenth volume of the *Cyclopedia of the Practice of Medicine* by Ziemssen.

Has it come to pass that the homœopathist is invited to resort to a palliative treatment—Amyl nitrite—not accepted by the better observers among our allopathic brethren, who even warn us against its general use? And what criticism is ready for our school when a *truly* scientific physician of any school reads on page 473, "I am satisfied there are cases closely simulating this disease which are wholly due to hysteria, uterine, and ovarian disease. In such cases Cimicifuga, Asafœtida, Lilium, and other utero-ovarian remedies prove useful. I have (says the writer of the paper) used in similar cases with brilliant results hypodermic injections of Morphia, Codea, and Atropia in minute quantities, and their effects were permanent, no recurrence appearing after the production of sound sleep." Based on the law of Homœopathy? What does H. R. Arndt, M. D., say to such an assertion? In disgust and sick, we hope to find something good in the work, and turn at random to page 669. There we find "Gallic acid in ten-grain doses, frequently repeated, Alum in two-grain doses, and the Acetate of lead are most reliable." Does H. R. Arndt call this modern Homœopathy?—a system of medicine based upon the law of Homœopathy? Woe to the young practitioner who has invested in this work. Woe to the victims who, under the expectation of homœopathic treatment, are dosed according to this marvelously pretentious work. Let them remember that the true guide to success as a true healer is to accept Hahnemann's teaching as it is found in his *Organon of the Healing Art* and accept as a true therapeutic guide his *Materia Medica Pura* and *Chronic Diseases*, as well as further late publications such as his *true followers* made and to beware of all false teachings not in harmony with the laws governing Homœopathy.

After due deliberation we come to the conclusion that the title-page of this work is misleading, that it will be found to be a fatal error by all intelligent men conversant with the laws governing Homœopathy, and that it should read "A system of medicine *ostensibly* based upon, but really *repudiating*, the law of Homœopathy."

A gentleman called for assistance on the 20th of September, 1885. For some six months his hair had been falling out in tufts on the back part of his scalp, leaving bald, smooth spots as large as a quarter of a dollar and smaller. He desired aid, so that he might finally not grow bald. About fifty years old, he had always enjoyed good health, and is at present perfectly well. No possible cause could be assigned for the peculiar falling out of the hair in spots. He was a strong homœopath, and I remembered him well, having some seven years previous waited on his six-year old boy in consultation with the late Dr. H. N. Guernsey, in a case of very malignant diphtheria. This case recovered fully under strictly homœopathic treatment. Again he wanted strictly homœopathic treatment. He received one single dose of Kali carb.$^{cm}$ (Fincke) on the 20th of September. He called again on the 12th day of October, and all the bald spots were covered with a new crop of hair growing splendidly.

*Comments.*—The case here related was by no means one of the many grave cases so often coming under treatment, but it was, nevertheless, a trying case. Apparently but one symptom presented itself, falling off of the hair in spots. The two best known remedies for this affliction are Hepar s. c. and Phosphorus. It was very desirable to detect, if possible, some other symptom which might guide the healer to find the truly homœopathic remedy. His hair had been exceedingly dry for some months, was not turning gray. Great dryness of the hair we find under Alumina and also under Kali carbonicum, and as Kali carb. has produced and healed falling off of the hair, we chose this remedy. With most happy results it was administered. The most difficult task for the careful physician is the finding of the symptoms—the examination of the sick; the more meagre the symptoms, the more difficult is the choice of the remedy. In this case the choice was Kali carbonicum. The second very important question is how to administer it. The safest way of administering a well-chosen remedy is to give one single dose of a well-developed potency without paying the least regard to a set of illogical, benighted pretenders who *a priori* guess that there is no medicinal virtue above the twelfth dilution, and while said benighted pretenders have NEVER yet presented a case illustrating their assumption that there is no curative power beyond the twelfth dilution, their assumptions must be ignored by all men who have made experiment honestly and come to the final conclusion that so far no limit to potentization can be set, as all potencies yet made have developed, if possible, increasing curative virtues. As to the single dose; if the similar remedy has been ascertained, the clinical experiment will teach every good observer that in most cases, acute and chronic, the single dose will develop all the curative powers necessary to restore health, and, to the contrary, a continuation of the proper remedy will just, in most cases, act detrimentally, causing accumulative medicinal effect on the already sick organism without giving the *vis medicatrix* time to overcome the diseased condition under the influence which a simple dose exerts on it. Such cures as are daily made by the true healer who relies on the law of the similars, the single remedy, and the single minimum dose, have been called, by the unfortunate illogical members of the profession "accidental cures." Maybe they are, but it is strange that they occur so very often, nay, regularly, under the same methods of practice. Cases of this kind have also connected with them their *lesson*. If our younger colleagues wish to save much anxiety and blundering, let them discard as false and deceptive guides all works on "practice," but hold fast to the teachings of Hahnemann and our *Materia Medica* as unerring guides in the discharge of the duties of a healer. Suppose just such a case as here related comes under the care of a younger colleague who has to establish a reputation for skill, will he find any advice or any help in any of the various works on "practice"? A shorter and safer method of finding the curative remedy is to consult our *Materia Medica*. There he will find the desired information, and there he will find in each individual case the truly homœopathic remedy. Where will he look in a book on practice for advice? Has this case above stated been labeled by the pathologist, and if not labeled where is the physician to look for advice? The promised fleshpots of Egypt will just vanish, and no comfort will come to him till he goes back to Samuel Hahnemann for advice and discards the pretenders. The publication of the results of the clinical experiments, the publication of cured cases, serves but one great purpose, viz.: to testify to the incontrovertible fact that the methods of Hahnemann and his directions *how* to apply the only law of cure, the law of the similars, for the cure of the sick, are corroborated by the experiment, by their practical applications in the science of therapeutics.

